# SARM1 deletion in parvalbumin neurons is associated with autism-like behaviors in mice

**DOI:** 10.1038/s41419-022-05083-2

**Published:** 2022-07-22

**Authors:** Ludan Xiang, Qian Wu, Huankun Sun, Xuemeng Miao, Zhaoting Lv, Huitao Liu, Lan Chen, Yanrou Gu, Jianjun Chen, Siyao Zhou, Huixia Jiang, Siyu Du, Yixin Zhou, Hui Dong, Yiren Fan, Shuangda Miao, Qi Lu, Liyun Chang, Hui Wang, Yi Lu, Xingxing Xu, Wei Wang, Zhihui Huang

**Affiliations:** 1 School of Mental Health, Wenzhou Medical University, Wenzhou, 325035 Zhejiang China; 2 Tongde Hospital of Zhejiang Province, Hangzhou, 310012 Zhejiang China; 3 Department of Orthopedics (Spine Surgery), The First Affiliated Hospital of Wenzhou Medical University, Wenzhou, 325000 Zhejiang China; 4 Department of Child Psychiatry, Shaoxing Seventh People’s Hospital, Shaoxing, 312000 Zhejiang China; 5 School of Basic Medical Sciences, Wenzhou Medical University, Wenzhou, 325035 Zhejiang China; 6 Zhejiang Provincial Clinical Research Center for Mental Disorders, The Affiliated Wenzhou Kangning Hospital, Wenzhou Medical University, Wenzhou, 325000 Zhejiang China; 7 Institute of Aging, Key Laboratory of Alzheimer’s Disease of Zhejiang Province, Wenzhou Medical University, Wenzhou, 325035 Zhejiang China; 8 College of Pharmacy, Hangzhou Normal University, Hangzhou, 311121 Zhejiang China

**Keywords:** Cell death in the nervous system, Autism spectrum disorders

## Abstract

Autism spectrum disorder (ASD), a group of neurodevelopmental disorder diseases, is characterized by social deficits, communication difficulties, and repetitive behaviors. Sterile alpha and TIR motif-containing 1 protein (SARM1) is known as an autism-associated protein and is enriched in brain tissue. Moreover, SARM1 knockdown mice exhibit autism-like behaviors. However, its specific mechanism in ASD pathogenesis remains unclear. Here we generated parvalbumin-positive interneurons (PVI)-specific conditional SARM1 knockout (SARM1^PV^-CKO) mice. SARM1^PV^-CKO male mice showed autism-like behaviors, such as mild social interaction deficits and repetitive behaviors. Moreover, we found that the expression level of parvalbumin was reduced in SARM1^PV^-CKO male mice, together with upregulated apoptosis-related proteins and more cleaved-caspase-3-positive PVIs, suggesting that knocking out SARM1 may cause a reduction in the number of PVIs due to apoptosis. Furthermore, the expression of c-fos was shown to increase in SARM1^PV^-CKO male mice, in combination with upregulation of excitatory postsynaptic proteins such as PSD-95 or neuroligin-1, indicating enhanced excitatory synaptic input in mutant mice. This notion was further supported by the partial rescue of autism-like behavior deficits by the administration of GABA receptor agonists in SARM1^PV^-CKO male mice. In conclusion, our findings suggest that SARM1 deficiency in PVIs may be involved in the pathogenesis of ASD.

## Introduction

Autism spectrum disorder (ASD), which refers to a group of neurodevelopmental disorders, is characterized by dysregulated social behavior and communication, as well as the presence of stereotyped or repetitive behaviors. In recent years, the prevalence of ASD has dramatically increased and been reported to be ~1% worldwide, similar to that in China [1, 2]. Many known autism risk genes are involved in multiple biological processes, such as synaptic signaling, protein translation regulation, and Wnt signaling. Unfortunately, such known pathogenic or risk genes account for only ~5% of all known ASD cases, leading to a lack of mechanism-based therapeutic options [3]. Therefore, the pathogenic mechanisms of ASD have not been fully elucidated, and it is important to identify more risk genes and the underlying mechanisms.

Sterile alpha and TIR motif-containing 1 (SARM1) protein, a member of the Toll/IL-1 receptor (TIR) domain protein family, was initially regarded as a negative regulator of innate immunity. Previously, the overexpression of SARM1 has been shown to inhibit LPS-mediated activation of TLR in human monocytic cells by interacting with TRIF and MyD88, two members of the TIR domain-containing adapter protein family [4]. However, SARM1, which is expressed in neuronal cells rather than microglia in the nervous system, seems to act as a proinflammatory factor in response to acute traumatic axonal injuries [5, 6]. Interestingly, studies have suggested that the SARM1 protein plays another important role as an NAD^+^ enzyme in the mitochondrial metabolism response to axonal injury, in turn causing axonal degeneration [7, 8]. These findings clearly suggest a pathological role of SARM1 in certain contexts. Given the enriched expression pattern of this protein in the central nervous system, this raises an important question about the physiological role of SARM1 in various higher brain functions such as cognition. Intriguingly, the *SARM1* gene is located on chromosome 17q11 in humans and was previously considered as a range of the ASD susceptibility genes (Auts6, OMIM% 609378) [9]. SARM1 has been reported to regulate neuronal morphogenesis at multiple levels, including through interactions with syndecan-2 to control dendritic arborization and regulate axonal outgrowth as well as neuronal polarity [10]. In addition, other studies have shown that reduced SARM1 expression in neuronal cells leads to changes in the postsynaptic protein composition, including NR1, NR2A, and shank proteins [11]. Furthermore, one previous study showed that knockdown of SARM1 in mice resulted in deficiencies in terms of social interactions [12]. However, the detailed functionality of SARM1 in ASD pathogenesis remains unclear.

Recent studies have shown that targeting ablation of many ASD-risk genes only to parvalbumin interneurons (PVIs) is sufficient to produce ASD-like phenotypes, indicating the important role of PVI dysfunction in the pathogenesis of ASD [13–15]. In our study, conditional SARM1 knockout (SARM1^PV^-CKO) mice were generated based on the high expression of SARM1 in PVIs. The epidemiological results showed that the incidence of autism in males is much higher than that in females, and the incidence ratio of males to females is approximately 4:1 [1]. Therefore, behavioral tests were performed on male mice throughout our study. Interestingly, SARM1^PV^-CKO male mice exhibited social-interaction deficiencies, as well as repetitive behaviors. Furthermore, we found that knocking out SARM1 may result in apoptosis in PVIs, as indicated by the reduced parvalbumin expression level and elevated level of cleaved caspase-3 within PVIs in SARM1^PV^-CKO mice. Given the effect of PVIs on the modulation of neuronal activity, the activation status of neurons was detected by immunostaining for c-fos protein in the brain. As expected, the level of c-fos protein in SARM1^PV^-CKO mice was significantly elevated compared with that in SARM1^f/f^ mice, along with the increased expression level of excitatory synapse protein postsynaptic density protein 95 (PSD-95) and neuroligin-1, indicating more excitatory synaptic input. Finally, the administration of GABA receptor agonists (muscimol and baclofen) partially rescued the autism-like behavior deficits in SARM1^PV^-CKO male mice. Taken together, these results suggest that the loss of SARM1 in PVIs may play a vital role in the pathological mechanisms of ASD by affecting PVI survival.

## Results

### SARM1 was upregulated in the brain tissue of several ASD model mice

**Fig. 1:**
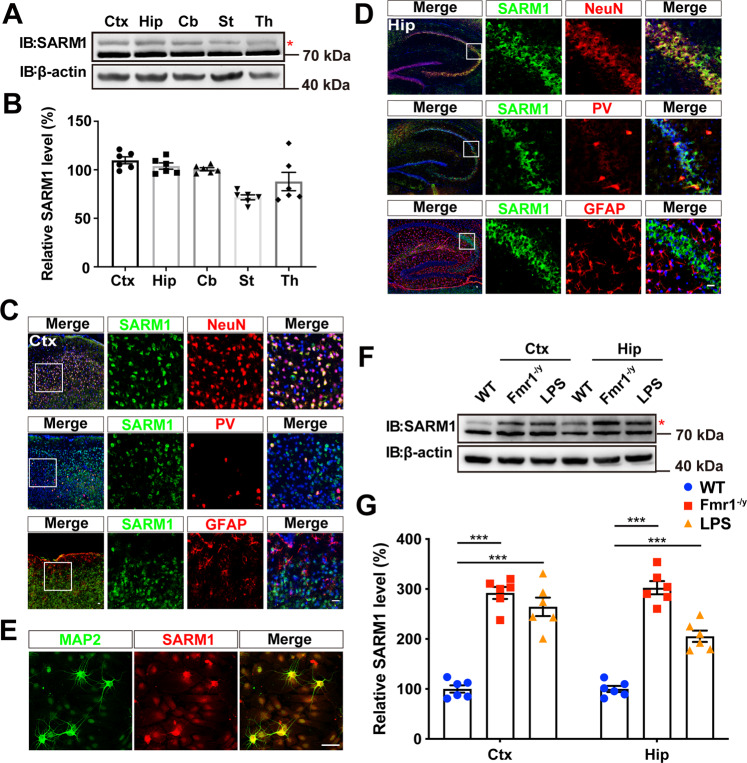
SARM1 was upregulated in the brain tissue of several ASD model mice. **A** Western blot analysis of the expression levels of SARM1 in the cortex (Ctx), hippocampus (Hip), cerebellum (Cb), striatum (St), and thalamus (Th) of 2-month-old male mice. **B** Quantitative analysis of SARM1 expression (*n* = 6 animals per group). **C**, **D** Double immunostaining of SARM1 (green) and NeuN (red) (upper panel), SARM1 (green) and PV (red) (middle panel), SARM1 (green) and GFAP (red) (lower panel) in the cortex (**C**) or hippocampus (**D**) of 2-month-old male mice. Images of selected regions (white squares) are shown at a higher magnification. **E** Double immunostaining of SARM1 (red) and MAP2 (green) in primary cultured neurons in vitro. **F** Western blot analysis of the expression levels of SARM1 in the cortex (Ctx) and hippocampus (Hip) of 2-month-old control mice, Fmr1 KO mice, and LPS-induced autism-like mouse models. **G** Quantitative analysis of SARM1 expression as shown in (**F**) (*n* = 6 animals per group). The red asterisk represents the specific band of SARM1. The density of the western blot bands was normalized to that of the β-actin protein. Data are presented as the mean ± SEM. Quantitative data were analyzed using the Student’s *t* test and compared to the control group. ***p < 0.001. Scale bars, 20 μm.

First, we examined the expression pattern of SARM1 protein in mouse brain tissues by using western blotting and immunostaining. We found that the SARM1 protein is widely expressed throughout the mouse brain (Fig. 1A, B). Coimmunostaining indicated that SARM1 protein was mainly expressed in neurons (including PV^+^ neurons) within the cortex and hippocampus, but at lower levels in GFAP-positive astrocytes (Fig. 1C, D). Furthermore, in cultured primary hippocampal neurons, we found that SARM1 was mainly expressed in soma and dendrites in cells by MAP2 costaining (Fig. 1E). Above, we confirmed that SARM1 was widely expressed in neurons in vivo and in vitro. To further evaluate the association between SARM1 and autism, we checked the expression level of SARM1 protein in two autism mouse models (*Fmr1*^*-/y*^, LPS-induced autistic mice). According to previous studies, *Fmr1*^*-/y*^ mice are a typical mouse model of genetic defects associated with autism, and LPS-induced autistic mice could represent an autism mouse model, which may be mainly caused by environmental factors via maternal immune activation (MIA) [16, 17]. As shown in Fig. 1F, G, the relative SARM1 protein levels in these two autism model mice were upregulated in the cerebral cortex and hippocampal regions. Taken together, our results implied the association of SARM1 with autism pathogenesis.

### SARM1^PV^-CKO male mice exhibited autism-like behaviors

**Fig. 2:**
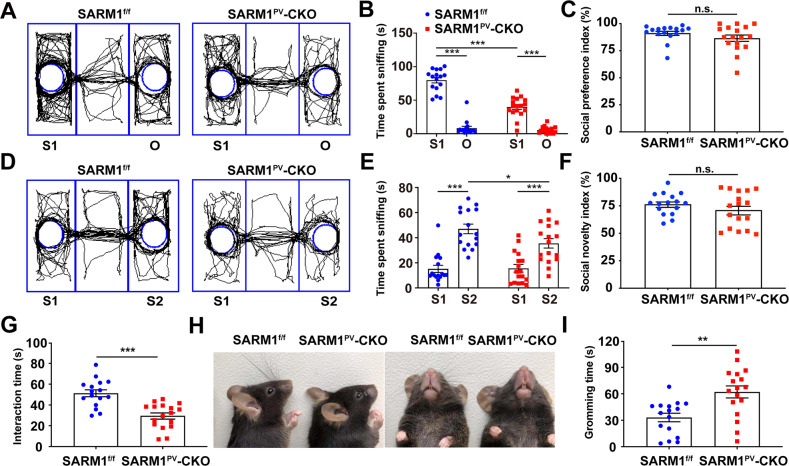
SARM1^PV^-CKO mice exhibited autism-like behaviors. **A**, **D** Schematic presentation of the trajectory diagram of 2-month-old SARM1^f/f^ (*n* = 16, left panel) and SARM1^PV^-CKO mice (*n* = 17, right panel) in the three-chamber social interaction test in session I (**A**) or session II (**D**). **B**, **C** Quantitative analysis of time spent sniffing in S1 or empty (O) in the three-chamber social interaction test (**B**) and social preference index (**C**) of 2-month-old male SARM1^f/f^ (*n* = 16 animals) or SARM1^PV^-CKO mice (*n* = 17 animals) as shown in (**A**). **E**, **F** Quantitative analysis of time spent sniffing in S1 or S2 in the three-chamber social interaction test (**E**) and social novelty index (**F**) of SARM1^f/f^ (*n* = 16 animals) or SARM1^PV^-CKO mice (*n* = 17 animals) as shown in (**D**). **G** Quantitative analysis of the interaction time spent in the direct social interaction test involving 2-month-old male SARM1^f/f^ (*n* = 16 animals) or SARM1^PV^-CKO mice (*n* = 17 animals). **H** The front (right panel) and side (left panel) view of the beard of 2-month-old male SARM1^f/f^ or SARM1^PV^-CKO mice. **I** Quantitative analysis of grooming time in repetitive behavior tests involving SARM1^f/f^ (*n* = 16 animals) or SARM1^PV^-CKO mice (*n* = 17 animals). Data are presented as the mean ± SEM. Quantitative data were analyzed using the Student’s *t* test and compared to the SARM1^f/f^ group. *p < 0.05, **p < 0.01, ***p < 0.001.

As mentioned previously, SARM1 knockdown mice show a deficiency in social interaction and cognitive flexibility [12], but are unable to provide more details, such as the functional-related cell-specificity of this protein. Based on the expression pattern of SARM1 and the pivotal role of PVIs in autism pathogenesis, we speculate that loss of SARM1 in PVIs may have an effect on social interaction. Therefore, PV neuron-specific conditional SARM1 knockout mice, SARM1^PV^-CKO mice, were established (Supplemental Figure S1A, B). Except for genotyping, the knockout of SARM1 in PVIs was confirmed by western blot and immunostaining (Supplemental Figure S1C–E). In general, SARM1^PV^-CKO mice did not differ in body weight from SARM1^f/f^ mice during development (Supplemental Figure S1F). Next, we performed autism-associated behavior tests on SARM1^PV^-CKO mice. In the three-chamber sociability test, SARM1^PV^-CKO mice exhibited a lower preference for exploring a stranger mouse compared to an inanimate object, as measured by the time spent sniffing a stranger mouse (S1) (Fig. 2A, B). After replacing the inanimate object with another stranger mouse (S2), SARM1^PV^-CKO mice exhibited less time spent sniffing to S2 than SARM1^f/f^ mice (Fig. 2D, E). In the direct social interaction test, where mice were allowed to freely interact with a moving stranger, SARM1^PV^-CKO mice spent less time interacting with the stranger than SARM1^f/f^ mice (Fig. 2G), also showing that SARM1^PV^-CKO mice had social deficiencies. The results of the olfactory habituation/dishabituation test showed no difference between SARM1^PV^-CKO and SARM1^f/f^ mice, ruling out the influence of olfactory disorders on social deficits (Supplemental Figure S2A). Stereotyped behavior, another core symptom of autism, is determined by the grooming time test. In comparison to SARM1^f/f^ mice, after exposure to a new home cage, SARM1^PV^-CKO mice exhibited enhanced self-grooming (Fig. 2H, I). In addition, SARM1^PV^-CKO mice did not exhibit cognitive dysfunction based on the results of the Barnes maze test (Supplemental Figure S2B, C). In conclusion, SARM1^PV^-CKO mice showed some autism-like deficiency, but no obvious cognitive dysfunction.

**Fig. 3:**
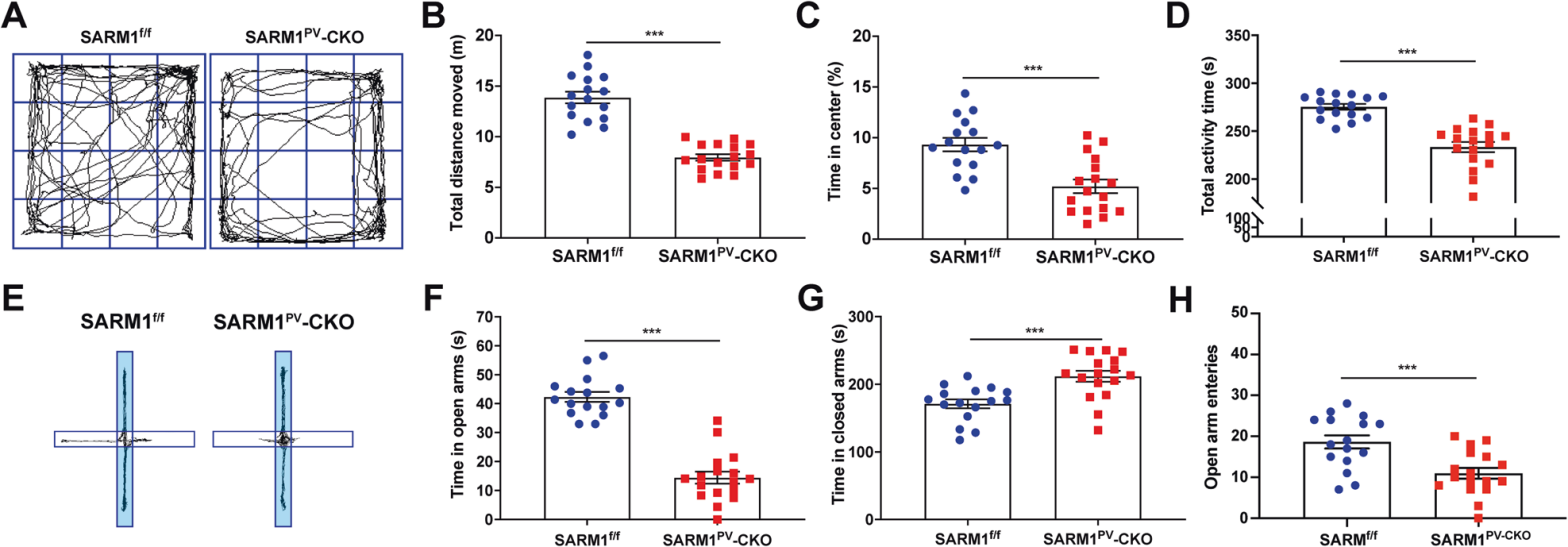
SARM1^PV^-CKO male mice exhibited obvious anxiety-like behaviors. **A** Schematic presentation of 2-month-old male SARM1^f/f^ (left panel) and SARM1^PV^-CKO (right panel) mice in the OFT. **B**–**D** Quantitative analysis of total distance moved (**B**), time spent in center (**C**), and total activity time (**D**) in OFT involving SARM1^f/f^ (*n* = 16) or SARM1^PV^-CKO mice (*n* = 17 animals) as shown in (**A**). **E** Schematic presentation of 2-month-old male SARM1^f/f^ (left panel) and SARM1^PV^-CKO (right panel) mice in the elevated plus maze tests. **F**–**H** Quantitative analysis of time spent in open arms (**F**) or closed arms (**G**) or open arm entries (**H**) in elevated plus maze tests involving SARM1^f/f^ (*n* = 16 animals) or SARM1^PV^-CKO mice (*n* = 17 animals). Quantitative data were analyzed using the Student’s *t* test and compared to the SARM1^f/f^ group. ***p < 0.001.

As reported previously, anxiety disorders are one of the most prevalent comorbidities in ASD [18]. To evaluate anxiety-associated behavior in SARM1^PV^-CKO mice, the open field test and the elevated plus maze test were performed. As a result, SARM1^PV^-CKO mice spent shorter time at the center of the open field, shorter time and fewer entries in the open arms of the elevated plus-maze, indicating that SARM1^PV^-CKO mice displayed anxiety-like behaviors (Fig. 3A–H). In summary, SARM1^PV^-CKO male mice fairly met autism-like features in behavior phenotypes.

### Parvalbumin was decreased in adult SARM1^PV^-CKO male mice

We next explored the cellular mechanisms by which the loss of the *Sarm1* gene in PVIs caused autism-like behavior features. To determine the effect of PVI-specific SARM1 knockout on the overall neuronal circuit, Nissl staining and immunohistochemistry of NeuN were applied to brain tissues from 2-month-old male mice. As shown in supplemental Figure S3, there were no significant differences in either the number or distribution of neurons between SARM1^f/f^ and SARM1^PV^-CKO mice in different brain regions. Further observation showed that the cortical cells presented triangular or round shapes and no obvious change or difference in cell size, shape or swelling degree between the two groups. In CA2/CA3 of the hippocampus, the cells seemed round and translucent without pyknotic nuclei, and the cell density was uniform, without nuclear debris and atrophic cell bodies. Therefore, conditional knockout of SARM1 in PVIs did not affect the number and distribution of neurons in the cortex and hippocampus.

**Fig. 4:**
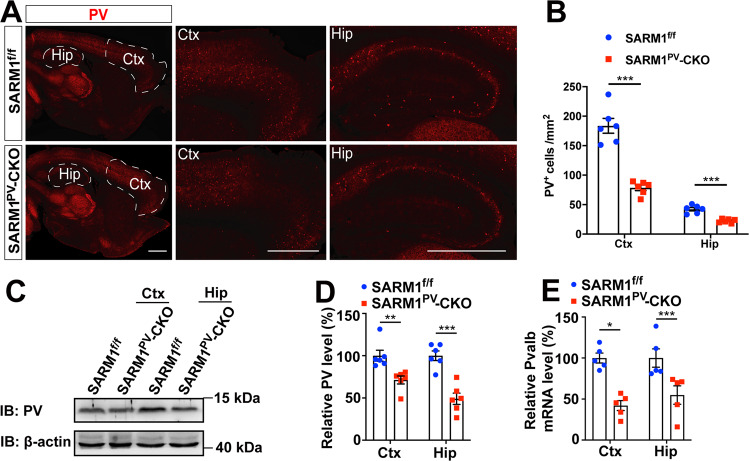
Parvalbumin decreased in adult SARM1^PV^-CKO male mice. **A** Immunostaining of PV (red) in the brain tissues of 2-month-old male SARM1^f/f^ and SARM1^PV^-CKO mice. Cortex and hippocampus images are shown at a higher magnification. **B** Quantitative analysis of the number of PV^+^ cells in the cortex and hippocampus per mm^2^ as shown in (**A**) (*n* = 6 animals per group). **C** Western blot analysis of the expression of PV in the cortex and hippocampus of 2-month-old male SARM1^f/f^ and SARM1^PV^-CKO mice. **D** Quantitative analysis of relative PV expression levels as shown in (**C**) (*n* = 6 animals per group). **E** qRT–PCR analysis of the mRNA expression levels of *Pvalb* in the cortex and hippocampus of 2-month-old male SARM1^f/f^ and SARM1^PV^-CKO mice (*n* = 5 animals per group). *β-actin* was used as housekeeping gene. The density of the western blot bands was normalized to that of the β-actin protein. Quantitative data were analyzed using the Student’s *t* test and compared to the control group. *p < 0.05, **p < 0.01, ***p < 0.001. Scale bars, 1 mm.

To determine whether knockout of SARM1 had a direct impact on PVIs, immunohistochemistry against parvalbumin was first performed. The number of parvalbumin-positive cells in the cortex and hippocampus was reduced in SARM1^PV^-CKO mice compared to that in SARM1^f/f^ mice (Fig. 4A, B). Moreover, as indicated by western blotting and qRT–PCR, parvalbumin expression in these regions was also decreased (Fig. 4C–E). Furthermore, PVIs were detected by double-label immunostaining for parvalbumin and Vicia Villosa Agglutinin (VVA), a lectin recognizing the specific extracellular matrix enwrapping PVIs [19], and the double-labeled cells were found to significantly reduce in the cortex of SARM1^PV^-CKO mice (Supplemental Figure S4A, B). Taken together, Thus, our results suggest the possibility that SARM1 knockout has an effect on proper PVI activity.

### Loss of parvalbumin-positive cells in SARM1^PV^-CKO male mice may be caused by XAF-1 activation-associated apoptosis

In principle, reduction in parvalbumin could result from the decreased number of PVIs, and the latter may be caused by decreased generation, more neuronal degeneration or both. It has been reported that parvalbumin is not expressed by interneurons in mice until the second postnatal week [20]. Therefore, to determine whether this reduction in PVIs was due to dysregulated cell generation, immunofluorescence staining of PVIs was performed at an early developmental stage. We found that there was no difference in the number of PVIs between SARM1^PV^-CKO mice and SARM1^f/f^ mice at P14 and P21 (Supplemental Figure S5A, B). This may imply that knocking out SARM1 may not impact the initial number of PVIs, which come from the differentiation of interneuron precursors.

Thus, it is more likely that the reduction in the number of PVIs in SARM1^PV^-CKO mice may be due to more cell damage. We cannot exclude the possibility that knocking out SARM1, once regarded as a negative regulator of the innate immune system, may trigger an autoimmune response, which in turn causes cell death. However, from the results of immunofluorescence staining of Iba1, GFAP, and CD45 in the brain tissues of mice, we found no apparent inflammation in the brain (Supplemental Figure S5C–I). In addition, based on recent reports of the function of SARM1 as NADase [8], we investigated the NAD^+^/NADH ratio by using the NADase assay, but there were no significant differences between the two groups (Supplemental Figure S5J).

**Fig. 5:**
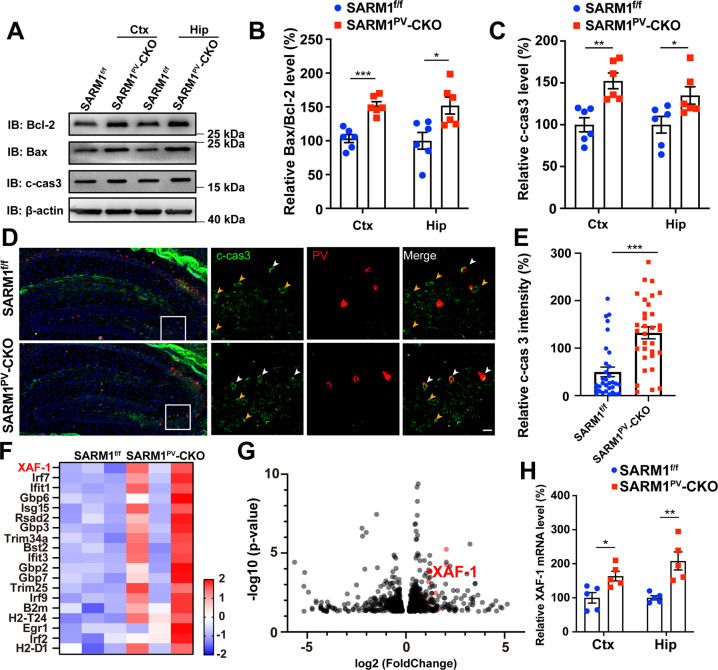
Loss of parvalbumin-positive cells in SARM1^PV^-CKO male mice may caused by XAF-1 activation-associated apoptosis. **A** Western blot analysis of the expression levels of Bcl-2, Bax, Cleaved caspase-3 (c-cas3) in the cortex and hippocampus of 2-month-old male SARM1^f/f^ and SARM1^PV^-CKO mice. **B**, **C** Quantitative analysis of the relative Bax/Bcl-2 (**B**) or c-cas3 (**C**) expression level as shown in (**A**) (*n* = 6 animals per group). **D** Immunostaining of c-cas3 (green) and PV (red) in the brain tissues at P25 of SARM1^f/f^ and SARM1^PV^-CKO mice. (**E**) Quantitative analysis of relative c-cas3 intensity in the hippocampus (*n* = 32 cells from 3 animals per group). **F** Heatmap of differential expressed IFN-stimulated genes (ISGs) from 2-month-old SARM1^f/f^ and SARM1^PV^-CKO mice brain (*n* = 3 animals per group). **G** Volcano plot of differential expressed genes (the red dots indicate ISGs) of apoptosis-related factor XAF-1 from 2-month-old SARM1^f/f^ and SARM1^PV^-CKO mice brain (*n* = 3 animals per group). **H** Quantitative analysis of relative XAF-1 mRNA expression level in the cortex and hippocampus of 2-month-old male SARM1^f/f^ and SARM1^PV^-CKO mice (*n* = 5 animals per group). *β-actin* was used as housekeeping gene. The density of the western blot bands was normalized to that of the β-actin protein. Data are presented as the mean ± SEM. Quantitative data were analyzed using the Student’s *t* test and compared to the control group. *p < 0.05, **p < 0.01, ***p < 0.001. Scale bars, 20 μm.

We next considered whether the reduced PVIs were due to increased apoptosis. First, the expression of apoptosis-related proteins was detected by western blot analysis. We found that the Bax/Bcl-2 ratio was increased in each brain area of the SARM1^PV^-CKO mice, and cleaved caspase-3 expression was also elevated in the cortex and hippocampus (Fig. 5A–C). Furthermore, cleaved caspase-3 intensity in PV^+^ neurons was found to be significantly increased in SARM1^PV^-CKO male mice (Fig. 5D, E). In summary, our results suggested that the reduction in PVIs may be due to the increased apoptosis. To further understand the underlying mechanisms, transcriptomic analysis of SARM1^f/f^ and SARM1^PV^-CKO male mouse brains was performed. We found that IFN-stimulated genes (ISGs)—including *XAF-1*, *Ifit1*, *Ifit3*, *Gbp2*, and *B2m*—were upregulated in SARM1^PV^-CKO male mouse brains, indicating that interferon signaling may have been activated (Fig. 5F). The upregulation of *XAF-1* mRNA was also confirmed in SARM1^PV^-CKO male mice by using a qRT–PCR assay (Fig. 5G, H). Notably, XIAP-associated factor 1 (XAF-1) was reported previously as a pro-apoptotic factor. Taken together, our results imply that SARM1 deletion in PVIs might increase neuronal cell apoptosis by upregulating the expression levels of pro-apoptotic factors such as XAF-1.

### Autism-like behaviors in SARM1^PV^-CKO mice may be due to the PVI dysfunction

**Fig. 6:**
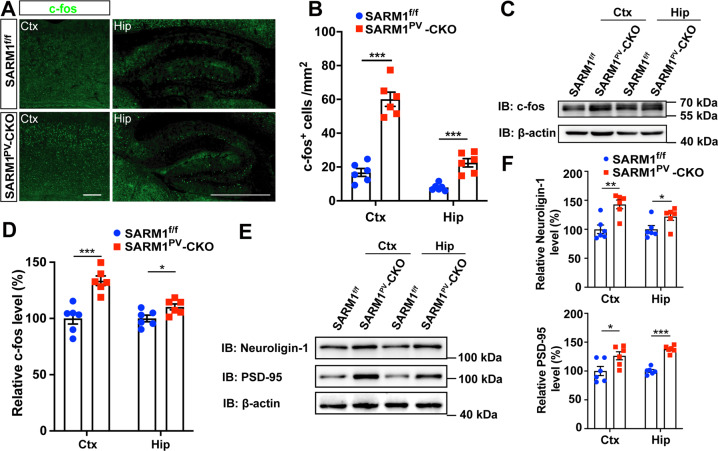
Autism-like behaviors in SARM1PV-CKO mice may be due to the PVI dysfunction . **A** Immunostaining of c-fos (green) in the brain of 2-month-old male SARM1^f/f^ and SARM1^PV^-CKO mice. Cortex and hippocampus images are shown at a higher magnification. **B** Quantitative analysis of the number of c-fos^+^ cells in the cortex and hippocampus per mm^2^ as shown in (**A**) (*n* = 6 animals per group). **C** Western blot analysis of the expression of c-fos in the cortex and hippocampus of 2-month-old male SARM1^f/f^ and SARM1^PV^-CKO mice. **D** Quantitative analysis of relative c-fos level as shown in (**C**) (*n* = 6 animals per group). **E** Western blot analysis of the expression levels of Neuronligin-1 and PSD-95 in the cortex and hippocampus of 2-month-old male SARM1^f/f^ and SARM1^PV^-CKO mice. **F** Quantitative analysis of the Neuroligin-1 and PSD-95 relative expression level as shown in (**E**) (*n* = 6 animals per group). The density of the western blot bands was normalized with that of β-actin protein. Data are presented as the mean ± SEM. Quantitative data were analyzed using the Student’s *t* test and compared to the control group. *p < 0.05, **p < 0.01, ***p < 0.001. Scale bars, 1 mm.

As shown above, our study revealed that knocking out SARM1 in PVI caused an autism-like behavioral phenotype and resulted in increased PVI apoptosis in mice. From these data, and according to previous literature, we hypothesized that the reduction of PVIs may relatively enhance excitatory synaptic input, which in turn caused autism-like behaviors in SARM1^PV^-CKO mice. To confirm our hypothesis, c-fos, serving as a marker of neuronal activity, was detected after social activation by immunohistochemical staining. As expected, the expression levels of c-fos in SARM1^PV^-CKO mice were found to be significantly elevated in the cortex and hippocampus in comparison to those of littermate control mice (Fig. 6A–D). In addition, we measured the expression levels of the excitatory postsynaptic proteins PSD-95 and neuroligin-1 and found that in the cortex and hippocampus, PSD-95 and neuroligin-1 were more highly expressed in SARM1^PV^-CKO male mice than in SARM1^f/f^ mice (Fig. 6E, F). All these results indicate that the reduction in PVIs in SARM1^PV^-CKO mice could increase excitatory synaptic input in neural circuits.

**Fig. 7:**
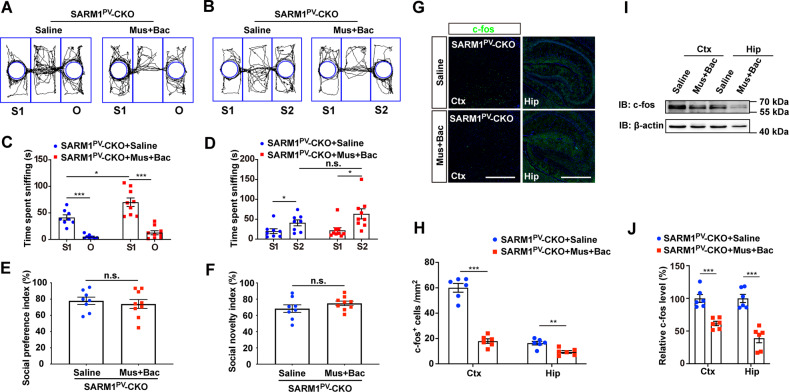
Increase of inhibitory activation partially rescued autism-like behavioral deficits in SARM1^PV^-CKO mice. **A**, **B** Schematic presentation of the trajectory of 2-month-old male SARM1^PV^-CKO mice after saline treatment (left panel), or SARM1^PV^-CKO mice after muscimol (Mus) and baclofen (Bac) treatment (right panel) in the three-chamber social interaction test in session I (**A**) or session II (**B**). **C**, **E** Quantitative analysis of time spent sniffing in S1 or empty (O) in the three-chamber social interaction test (**C**) or social preference index (**E**) of SARM1^PV^-CKO mice with saline treatment (*n* = 8 animals per group), or SARM1^PV^-CKO mice with muscimol (Mus) and baclofen (Bac) treatment (*n* = 9 animals per group) as shown in (**A**). **D**, **F** Quantitative analysis of time spent sniffing in S1 or S2 in the three-chamber social interaction test (**D**) or social novelty index of (**F**) 2-month-old SARM1^PV^-CKO mice after saline treatment (*n* = 8 animals per group) or SARM1^PV^-CKO mice after muscimol (Mus) and baclofen (Bac) treatment (*n* = 9 animals per group) as shown in (**B**). **G** Immunostaining of c-fos (green) in the cortex and hippocampus of 2-month-old male SARM1^PV^-CKO mice with or without drugs treatment. (**H**) Quantitative analysis of the number of c-fos^+^ cells in the cortex and hippocampus per mm^2^ as shown in (**G**) (*n* = 6 animals per group). **I** Western blot analysis of the expression levels of c-fos in the cortex and hippocampus of 2-month-old male SARM1^PV^-CKO mice with or without treatment. **J** Quantitative analysis of relative c-fos levels as shown in (**I**) (*n* = 6 animals per group). The density of the western blot bands was normalized to that of the β-actin protein. Data are presented as the mean ± SEM. Quantitative data were analyzed using the Student’s *t* test and compared to the control group. *p < 0.05, **p < 0.01, ***p < 0.001. Scale bars, 1 mm.

Inferring from our previous experimental results, autism-like behavior could be improved by activating GABA receptors, which could increase inhibitory output. Therefore, GABA receptor agonists were injected into the lateral ventricles of SARM1^PV^-CKO male mice. Additionally, the same amount of normal saline was injected into the control mice. Subsequently, three-chamber tests were performed on the treated mice, and it was found that sociality was partially improved in the drug treatment group (Fig. 7A–D). However, treatment with GABA receptor agonists did not have an effect on social novelty in SARM1^PV^-CKO male mice. After drug treatment, the expression of c-fos was obviously decreased in SARM1^PV^-CKO male mice as shown in Fig. 7G–J. Overall, the results of the above rescue experiments confirmed our hypothesis.

## Discussion

In this study, we showed that selectively knocking out SARM1 in PVIs causes autism-like behavior, including social deficits, repetitive grooming, and comorbidity with anxiety disorder in mice. Furthermore, we provide insight into the mechanism underlying the occurrence of these phenotypes [1]. Selectively knocking out SARM1 reduces the number of PVIs in the cortex, which might result from the increased apoptosis triggered by upregulation of pro-apoptotic factors such as XAF-1 [2]. Enhanced excitatory synaptic input, induced by the reduction of PVIs, leads to autism-like phenotypes. Taken together, our results indicate that the expression of SARM1 in PVIs plays a vital role in the pathogenesis of autism spectrum disorder.

Recently, the SARM1 protein has been highlighted as a promising target in multiple neurological diseases such as spinal cord injury, traumatic brain injury and sympathetic neuropathy [6, 21, 22]. The functional role of SARM1 as a proinflammatory factor and NADase is involved in the pathogenesis mechanism underlying these diseases. However, it remains insufficient given the physiological role of SARM1 in advanced brain function. Previously, one study reported that SARM1 was found to be reduced in the autopsy brain tissue of ASD patients, although the sample size is a limitation in that study [23]. In our study, upregulation of SARM1 protein was found in the brain tissue of two autism mouse models, Fmr1 KO mice and an LPS-induced MIA mouse model, which could represent a genetic model and an environmental model of ASD. In Fmr1 KO mice, loss of FMRP, an RNA-binding protein encoded by Fmr1, can cause translational upregulation of FMRP target genes including some ASD-risk genes [24, 25]. Sarm1 mRNA might be an unidentified target for FMRP protein as predicted previously [26]. This may explain the upregulation of SARM1 in Fmr1 KO mice. For LPS-induced autism mice, it has been reported that the expression level of SARM1 is upregulated in cultured neuronal cells treated with LPS [27]. Thus, it is plausible that SARM1 can be upregulated in the brains of offspring mice exposed to LPS. However, our findings allow us to provide another explanation, in which upregulation of SARM1 could be a compensator effect. Either in MIA model or Fmr1 KO mice, PVIs intend to recover their disrupted activity by upregulating the expression level of SARM1. In any case, our results suggest the significant correlation between SARM1 protein and ASD.

As mentioned above, SARM1 knockdown mice exhibited cognitive inflexibility and social deficiencies [12]. SARM1 has been reported to regulate neuronal morphogenesis by regulating dendritic arborization and axonal outgrowth [10]. Many ASD-risk genes are involved in synaptic connections by regulating pre- or postsynaptic structures during development, encouraging the possibility that neuronal-specific conditional SARM1 knockout may show autism-like phenotypes. As hoped, SARM1^PV^-CKO male mice show autism-like phenotypes and comorbidity with anxiety disorder in mice. Certainly, no difference in the social preference index suggests that impairment in social interaction in SARM1^PV^-CKO mice is not comparable to that of typical autism mouse models, such as Shank3 KO mice or Fmr1 KO mice. Given the highly heterogeneous nature of ASD, we inferred that SARM1 deficiency may be a relatively weak susceptibility factor for ASD.

We also observed that the expression of parvalbumin was reduced in the brain tissue of SARM1^PV^-CKO mice. Later, upregulation of apoptosis-related proteins and increased cleaved caspase-3 costained with PVIs were found in SARM1^PV^-CKO mice. Taken together, our results suggest that knocking out SARM1 likely causes more apoptosis in PVIs, which in turn leads to a reduction in PVIs. This finding was unexpected and surprising. According to previous studies, SARM1 may act as an NADase to cause neuronal cell death until it is activated in the appropriate context, such as axotomy [28]. Normally, the loss of SARM1 may have a protective effect against neuronal damage. Although XAF-1, a pro-apoptotic molecule, was found to be upregulated in SARM1^PV^-CKO mice, the mechanism by which a lack of SARM1 results in the upregulation of pro-apoptotic factors remains obscure. In fact, a recent study showed that SARM1 can be activated as an NADase in intact neuronal cells in response to alterations in the ratio between NMN and NAD^+^ [29]. This result likely suggests that SARM1 may act as a brake on mitochondrial metabolism under physiological conditions. In addition, PV neurons show the high energy expenditure during gamma oscillations [30]. Thus, loss of SARM1 may cause metabolic stress in PVIs, which in turn triggers apoptosis. In the future, our conjecture needs to be further confirmed.

In the last part of the results, we found that c-fos protein level were upregulated in multiple brain regions in SARM1^PV^-CKO mice, as well as the upregulation of PSD-95 and neuroligin-1, the biomarkers for excitatory synapse, which may suggest higher neuronal excitability in SARM1^PV^-CKO male mice. We noticed that the increased c-fos expression in SARM1^PV^-CKO mice after social stimulation is inconsistent with previous studies, which showed a downregulation of c-fos expression in some ASD mouse model [31]. However, c-fos levels may not always be consistent to the active behaviors in ASD model mouse. For example, compared with the WT mice, Fmr1 KO mice displayed hyperactivity and anxiety phenotype [32], however, the basal expression level of c-fos is decreased in OFC and mPFC of Fmr1 KO mice [33]. In addition, Cntnap2^−/−^ mice showed impaired texture discrimination through whiskers and showed an increase in c-fos expression following stimulated by whisker [34]. In our model, the upregulation of c-fos indicated higher excitation in general neuronal cells, but not in behaviors which might be regulated by multiple brain regions and more complex mechanisms. Furthermore, we observed that the social behavior of SARM1^PV^-CKO mice was partially restored following the decrease of neuronal excitability indicated by downregulated c-fos protein level, by the administration of GABA receptor agonists, which further support our deduction that neuronal excitability input may be enhanced in SARM1^PV^-CKO male mice.

In conclusion, conditional knockout of SARM1 in PVIs resulted in the autism-like phenotype of SARM1^PV^-CKO mice by affecting the number or functions of PVIs. There were also several limitations of this study that should be addressed. The mechanism through which SARM1 deficiency leads to apoptosis of PVIs is not fully understood. Moreover, enhanced excitatory synaptic input was inferred from dysfunction of PVIs and upregulated c-fos expression but was not confirmed by electrophysiological experiments. Furthermore, the role of SARM1 in other types of neuronal cells should be further investigated. Additional experiments are needed to determine the role of SARM1 in maintaining the function of PVIs and to expand the potential of SARM1 in ASD treatment.

## Materials and methods

### Animals

Wild-type (WT, C57BL/6 strain), Fmr1 KO (B6. 129P2-*Fmr1*^*tm1Cgr*^/J, the Jackson Laboratory), PV-Cre (B6. 129P2-*Pvalb*^*tm1(cre)Arbr*^/J, the Jackson Laboratory), SARM1^flox/flox^ (SARM1^f/f^) (Shanghai Model Organisms Center, Inc.) mice were purchased and bred in the animal facility at Wenzhou Medical University. All animals were provided with adequate food and water in a 14 h light/10 h dark cycle environment. PV neuron-specific conditional SARM1 knockout mice (SARM1^PV^-CKO) were generated by crossing PV-Cre mice with SARM1^f/f^ mice. All mice were maintained on a C57BL/6 background. SARM1^PV^-CKO mice were genotyped by polymerase chain reaction using the primers SARM1 floxed (500 bp) and WT (466 bp) version of the SARM1 allele, forward, 5′-AGCAACAAGCACTCTGAATGG-3′, reverse, 5′-AGATCACGCCTAGACCGATG-3′; for the PV-Cre allele (163 bp), forward, 5′-AAATGCTTCTGTCCGTTTGC-3′, reverse, 5′-ATGTTTAGCTGGCCCAAATG-3′; and for WT without PV-Cre (500 bp), forward, 5′-CAGAGCAGGCATGGTGACTA-3′, reverse, 5′-AGTACCAAGCAGGCAGGAGA-3′. For the preparation of MIA autism model mice, pregnant WT mice were randomly divided into two groups. The LPS-treated group received intraperitoneal LPS (L3129, Sigma) administration at a dose of 75 µg/kg on day 11.5 and day 12 of embryo growth, while the control group received an equivalent volume of saline. The 2-month-old male offspring were subjected to social interaction and grooming tests to validate the experimental model. All animal studies were carried out with the approval of the Animal Care and Use Committee of Wenzhou Medical University in Wenzhou (IACUC no: 2018-666242).

### Primary mouse neuron culture

The hippocampus was isolated from 17- to 18-day mouse embryos and dissected in a 60 mm petri dish with ice-cold 1× HBSS (without CaCl_2_ and MgCl_2_, Gibco). The cells were digested by incubating in 0.25% trypsin (Gibco) for 15 min at 37 °C. Then, the hippocampal tissues were washed and triturated into a single cell suspension in trituration medium containing 5% bovine serum (Gibco), 1% penicillin-streptomycin antibiotics, and 94% DMEM high glucose medium (Gibco). Cultured cells were plated on the poly-D-lysine (Sigma)-coated coverslips in 6-well plates. Finally, the trituration medium in the culture plate was completely replaced with neural basal working medium containing 1% penicillin-streptomycin antibiotics, 1× B27 supplement, 500 µM GlutaMax, and 98% neuron basal medium.

### Stereotaxic surgery and drug injection

Male 2-month-old SARM1^PV^-CKO mice were randomly divided into two groups and then stereotaxic surgery was performed as previously described [35]. These mice were anesthetized with an intraperitoneal injection of 1.25% tribromoethanol (0.2 ml/10 g), and immobilized on a stereotaxic apparatus (Stoelting, 51500). An incision was made in the skin from the middle region of the eyes to the ears. The skin was held with forceps, and the periosteum was gently removed. The skull was exposed, and a hole was made using a microdrill (RWD, code 78001). The gauge guide cannula was implanted into the lateral ventricle (AP −0.2 mm, ML 1.0 mm, DV 2.5 mm). The cannula was fixed to the skull with dental acrylic. After surgery, mice were housed individually and allowed to recover for 7 d. For drug injection, a stainless-steel injector connected to a 5-μl syringe was inserted into the guide cannula and extended 1 mm beyond the tip. The administration of the chemicals was performed on the basis of previous studies [36]. For the treatment group, 62.5 ng muscimol (Mus) (5060440001, MCE) and 62.5 ng baclofen (Bac) (B5399, Sigma) in 2 μl saline were infused over 5 min and the injector was kept for another 5 min. For the control group, an equal volume of saline was injected. After 7 h, when the effect of Mus+Bac drugs on the locomotor activity of mice could not be observed, a three-chamber social test was performed.

### Mouse behavioral tests

Since the incidence of autism in males is ~4-fold that in females, all behavioral tests were assayed on 2-month-old male mice during the light phase [37]. For SARM1^f/f^ and SARM1^PV^-CKO mice, the behavioral assays were performed in the following order: open field test, elevated plus-maze test, Barnes maze test, olfactory habituation/dishabituation test, direct social interaction, grooming test, and three-chamber test. For the SARM1^PV^-CKO Mus+Bac drug-treated group and control group, a three-chamber social test was performed. The human observers were blinded to the group allocation.

#### Three-chamber social test

This test, consisting of three 10-min sessions, was performed to evaluate the social abilities of mice as described in a prior study [38]. Briefly, mice were first habituated for 10 min in an empty plexiglass arena (60 × 40 × 23 cm size), which was divided into three interconnected chambers (left, center, right). Sociability was evaluated during the second 10-min period in which the subject could interact with an empty wire cup (Object, O) or a wire cup containing an age- and sex-matched stranger conspecific (Stranger 1, S1). The interaction time was determined by measuring the time that the subject mouse spent sniffing Object or Stranger 1. The position of the empty cup/stranger mouse in the left or right chamber during the sociability period was counterbalanced between trials to avoid bias. Preference for social novelty was assayed in a third 10-min period, by introducing a second stranger mouse (Stranger 2, S2) into the previously empty wire cup. All time subjects spent interacting with the object, stranger 1 or stranger 2 was recorded by an overhead camera and measured using the automated DigBehv software (Shanghai Jiliang Software Technology Co., Ltd.) by trained, independent observers.

#### Direct social interaction test

Mice were introduced into a cage with a 1-cm clean corncob and allowed to acclimatize for 5 min. After habituation, a stranger mouse with the same age, size, and sex was introduced into the cage. The mice could freely engage socially for 10 min and the cumulative time spent interacting with the stranger mouse was determined. Interaction behaviors were defined as nose-to-anogenital sniffing, nose-to-nose sniffing, and social grooming.

#### Self-grooming test

Mice were individually placed in a new empty cage. Each mouse was habituated to the empty cage for 10 min, and then the cumulative time spent grooming all body regions over 10 min was determined.

#### Olfactory habituation/dishabituation test

The olfactory habituation/dishabituation test was performed as described in a previous study [39]. The mice were allowed to acclimate for 30 min in the test cage with a clean dry applicator, which was inserted on the cage lid. Then, applicators with different odors were presented sequentially in the following order: distilled water, almond odor, banana odor, social odor 1, and social odor 2. The natural odors of bitter almond (McCormick, Hunt Valley) and banana extracts (McCormick, Hunt Valley) were diluted to 1:100 in distilled water. The social odor was prepared by swiping a cotton swab in the dirty cages of unfamiliar males before each trial. For each odor, three consecutive tests were performed for each odor. Each test involved 2 min of odor stimulation. The time interval between each trial was less than 1 min. The time spent sniffing the applicator in each trial was recorded.

#### Barnes maze test

To assess spatial learning and memory impairments in mice, the Barnes maze test was used as described in a previous study [40]. Mice learned to find an escape chamber through 1 of 20 holes (5-cm diameter, 5 cm from perimeter) below an elevated, brightly lit, and noisy platform using visual cues placed around the room. On the first day, the 2-month-old mice were trained to locate an escape box based on distal visual cues to escape from a platform with harsh light and strong wind. The mice received two trials per day (15-min intertrial interval) for five training days. In each trial, mice remained in the escape box for 60 s before being returned to their home cages. On day 9, a probe trial was performed. The number of errors and the latency to find the target hole (amount of time elapsed before nose poke over target hole) were measured.

#### Open field test

The autonomous behavior, exploratory behavior, and tension of the experimental animals were evaluated by open field tests in various new environments [41]. In brief, the 2-month-old male mice were placed in a chamber (40 × 40 × 37 cm size), and mouse movement was monitored for 5 min. The illuminance was simulated by 4 energy-saving lamps on both sides of the wall that emitted ~200 lux illumination, and the background noise of the laboratory was controlled below 65 dB. In the analysis, the center area of the chamber was defined as the 20 × 20 cm zone in the center. The total distance and time spent in the central area were measured using DigBehv software (Shanghai Jiliang Software Technology Co., Ltd.).

#### Elevated plus-maze test

This test utilized a simple, rapid, and reproducible model for evaluating anxiety behaviors in animals. In brief, 2-month-old male mice were introduced to the center of the apparatus and allowed to freely explore in a quiet environment for 5 min. The time spent and the number of entries in each arm were measured using the DigBehv software (Shanghai Jiliang Software Technology Co., Ltd.).

### Western blot

Total protein from mouse brain tissues was extracted using RIPA buffer containing protease inhibitor cocktail (Beyotime). Tissue lysates were sonicated, and denatured protein was separated by SDS–PAGE (8–12%) and transferred into nitrocellulose (G9944566, GE Amersham) or polyvinylidene fluoride membranes (ISEQ10100, Millipore). Membranes were blocked with 5% skimmed milk for 1.5 h and incubated with primary antibodies at 4 °C overnight, followed by incubation with secondary antibodies at room temperature for 1 h. The primary antibodies and secondary antibodies used in this study are shown in Table 1. Finally, protein bands were developed using ECL (Bio-Rad), and image analysis was conducted using ImageJ. Data were normalized to β-actin.

**Table 1:** Antibody list.

Antibodies (dilution)	Source	Identifier
*For western blot*		
rabbit anti-SARM1 (1:1000)	GeneTex	GTX131411
rabbit anti-c-fos (1:2000)	Abcam	ab190289
rabbit anti-Bcl-2 (1:1000)	CST	D17C4
rabbit anti-Bax (1:1000)	Abclonal	A0207
rabbit anti-Cleaved caspase-3 (1:1000)	CST	9664
rabbit anti-PV (1:1000)	Swant	PV27
rabbit anti-PSD-95 (1:1000)	CST	D74D3
mouse anti-neuroligin-1 (1:1000)	Santa Cruz	sc-365110
mouse anti-β-actin (1:10,000)	Sigma	A5316
HRP-conjugated Affinipure Goat Anti-Mouse IgG(H + L)	Proteintech	SA00001-1
HRP-conjugated Affinipure Goat Anti-Rabbit IgG(H + L)	Proteintech	SA00001-2
*For immunohistochemistry*		
rabbit anti-SARM1 (1:500)	Abcam	ab226930
mouse anti-MAP2 (1:500)	Abcam	ab5392
mouse anti-NeuN (1:500)	Millipore	MAB377
rabbit anti-NeuN (1:500)	Proteintech	66836-1-Ig
rabbit anti-PV (1:500)	Swant	PV27
mouse anti-PV (1:500)	Swant	235
rabbit anti-Cleaved caspase-3 (1:50)	CST	9664
rabbit anti-c-fos (1:200)	Abcam	ab190289
mouse anti-GFAP (1:500)	Millipore	MAB360
goat anti-Iba1 (1:500)	Abcam	ab5076
Goat anti-Rabbit IgG (H + L) Highly Cross-Adsorbed Secondary Antibody, Alexa Fluor Plus 488 (1:1000)	Invitrogen	A32731
Goat anti-Rabbit IgG (H + L) Highly Cross-Adsorbed Secondary Antibody, Alexa Fluor Plus 555 (1:1000)	Invitrogen	A32732
Goat anti-Mouse IgG (H + L) Highly Cross-Adsorbed Secondary Antibody, Alexa Fluor Plus 488 (1:1000)	Invitrogen	A32723
Goat anti-Mouse IgG (H + L) Highly Cross-Adsorbed Secondary Antibody, Alexa Fluor Plus 555 (1:1000)	Invitrogen	A32727

### Immunofluorescence and immunohistochemistry staining

For cultured cells, cells seeded on coverslips were fixed with 4% paraformaldehyde (PFA) in PBS for 15 min, permeabilized using 0.1% Triton X-100 in PBS for 10 min, and blocked with 5% bovine serum albumin (BSA) in PBS at room temperature for 1 h. Then, the cells were incubated with primary antibodies at 4 °C overnight, rinsed three times in PBS, and incubated with secondary antibodies at room temperature for 1 h. The antibodies used are listed in Table 1. After rinsing three times in PBS, the coverslips were mounted with mounting media with DAPI (Solarbio).

For tissue histology, brain tissues were fixed with 4% PFA for 48 h, dehydrated using a 30% sucrose solution for another 48 h, and embedded with Tissue-Tek OCT compound. In our experiments, six or seven pairs of mice were generally used for morphological examination. Referring to the mouse brain atlas, sagittal sections were cut from 0.24 mm lateral to the bregma point at a thickness of 20 μm. More than three parallel sections per mouse were used for analyses. The tissue sections were washed with PBS and fixed with 4% PFA for 30 min. For antigen retrieval, slides were incubated in sodium citrate antigen retrieval buffer for 30 min at 90 °C, then slides were blocked and permeabilized with 5% BSA plus 0.3% Triton X-100 in PBS for 1 h at room temperature and incubated overnight with primary antibodies at 4 °C. Slides were washed three times in PBS and incubated with corresponding secondary antibodies at room temperature for 1 h. The primary and secondary antibodies used are shown in Table 1. For the double staining of VVA and PV, 10 μg/ml biotinylated-VVA (B-1235-2, vector laboratories) and rabbit anti-PV primary antibody (see Table 1) were diluted in 5% BSA. After incubated overnight at 4 °C, sections were washed and incubated in corresponding secondary antibody (see Table 1) diluted in 5% BSA at room temperature for 1 h and then in ABC reagent in Vectastain Elite ABC-HRP Kit (PK-6200, Vector laboratories) for 30 min. Sections were washed three times in PBS and then incubated in 3,3′-diaminobenzidine (DAB) peroxidase (Zhongshan Jinqiao Biotechnology) for 30 s. After being washed several times, the coverslips were mounted with mounting media with DAPI (Solarbio). Images were collected by confocal or fluorescence microscopy (Ci-L, Nikon) using a multitrack configuration, and processed using ImageJ.

### Nissl staining

Nissl staining was performed as described in a previous study [42]. Brain tissues were separated as mentioned above. The slides were washed three times with PBS, then the sections were incubated with 0.1% cresyl violet for 6 min at room temperature, and then differentiated in 95% and 100% ethanol, followed by dehydration in 100% ethanol. Finally, they were cleared in xylene, and mounted with neutral resins. Images were analyzed using the ImageJ software.

### RNA sequencing and functional enrichment analysis

Total RNA was extracted from the control and mutant mouse brain tissue using the RNAiso Plus (TRIzol) reagent (T9108, Takara). The extracted RNA was sequenced at Beijing Novogene Co., Ltd. Next, the DESeq2 software (1.16.1) was used to evaluate differences in expression between the two mouse groups. From this analysis, genes with adjusted *p* < 0.05 values were differentially expressed. Differential expression analysis was performed using edgeR (3.18.1) software. Moreover, the Cluster Profiler R software was used to evaluate the statistical enrichment of differentially expressed genes in the GO terms and KEGG pathways. The heatmap and volcano map of differentially expressed genes were generated using GraphPad Prism version 8.

### RNA extraction and quantitative real-time PCR (qRT–PCR)

RNA extraction from tissues was performed using RNAiso Plus (TRIzol) (T9108, Takara). The concentration of the extracted RNA was measured using UV spectroscopy, after which 1 μg was reverse transcribed using the TransScript® All-in-One First-Strand cDNA Synthesis SuperMix (One-Step gDNA Removal) kit (AT341, TransGen Biotech). The mRNA expression levels of *Pvalb* and *Xaf-1* were quantified by TransStart^®^ Top Green Supermax (AQ131, TransGen Biotech) on the Real-Time PCR Detection System (CFX96, Bio-Rad). The primers (Sangon Biotech) were as follows: *Pvalb* primer (*T*_*m*_ = 85.7 °C), forward, 5′-TGTCGATGACAGACGTGCTC-3′ and reverse, 5′-TTCTTCAACCCCAATCTTGC-3′; *Xaf-1* primer (*T*_*m*_ = 85.7 °C): forward, 5′-GAAGCTTGACCATGGAGGCT-3′, and reverse, 5′-GGTGCACAACTTCCATGTGCT-3′ [43]; and *β-actin* primer (*T*_*m*_ = 86.9 °C): forward, 5′-AAGGAAGGCTGGAAAAGAGC-3′ and reverse 5′-GCTACAGCTTCACCACCACA-3′. Thermal cycling conditions were as follows: 94 °C for 30 s, followed by 40 cycles of 94 °C for 5 s and 60 °C for 30 s in a 20-μl reaction volume. Multiple changes in gene expression were calculated by the 2^−ΔΔCt^ method, with β-actin as an internal control.

### NAD^+^/NADH test

The NAD^+^/NADH ratio in mouse cortexes was detected using an NAD^+^/NADH Assay Kit (colorimetric) (ab65348, Abcam). In brief, the total NAD^+^ and NADH (NADt) were extracted from tissues using the NADH/NAD extraction buffer. To detect NADH, the samples were heated to 60 °C for 30 min and NAD^+^ was decomposed. The samples were mixed with 100 μL of the Reaction Mix or 100 μL of the Background Reaction Mix to promote a reaction. After 5 min, 10 μL of NADH developer per well was added to the reaction mixtures, followed by incubation at room temperature. During the reaction, multiple readings were taken between 1 and 4 h at OD 450 nm using a plate reader. The NAD^+^/NADH ratio was calculated as follows: NAD^+^/NADH ratio = (NADt-NADH)/NADH.

### Statistical analysis

The sample size was chosen based on previous experience with behavior tests and studies previously published. All data are reported as the mean ± SEM from at least three independent experiments. To facilitate a comparison between different groups, the Student’s *t* test or ANOVA analysis with Bonferroni post-tests was used. Statistical significance was set at *p* < 0.05.

## Supplementary information

Supplemental Figures

Behavior tests

Original western blots

Reproducibility Checklist

## Data Availability

The data that support the finding of this study are available upon request from the corresponding author.
