# Supplementary material for: SARM1 deletion in parvalbumin neurons is associated with autism-like behaviors in mice
Source: Cell Death Dis. 2022 Jul 22;13(7):638. doi: 10.1038/s41419-022-05083-2 (PMC9307765; doi:10.1038/s41419-022-05083-2)
Supplement: Supplementary file 1 — Supplemental Figures [file 41419_2022_5083_MOESM1_ESM.docx]

**Supplemental Figures**

**
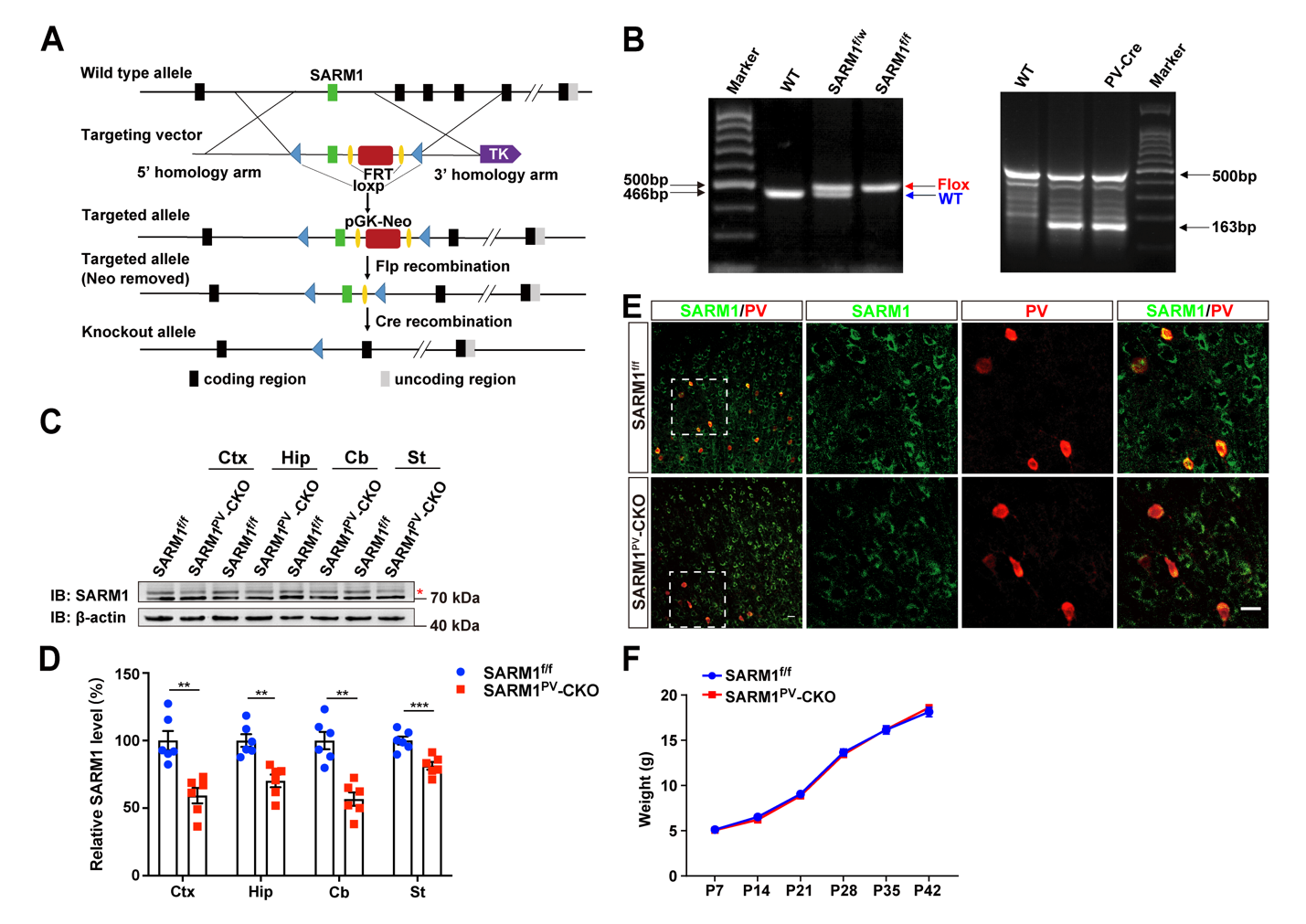
**

**Figure S1. Identification of SARM1^PV^-CKO mice.** (A) Schematic presentation of design strategy for SARM1 gene conditional knockout flox mice. (B) Genotyping was performed by agarose gel electrophoresis (SARM1^f/w^, SARM1^f/f^ mice were shown on the left panel, while PV-Cre mice on the right panel). (C) Western blot analysis of the expression levels of SARM1 in the cortex, hippocampus, cerebellum, and striatum of 2-month-old male SARM1^f/f^ and SARM1^PV^-CKO mice. (D) Quantitative analysis of the relative SARM1 levels as shown in (C) (n=9 animals per group). (E) Immunostaining of SARM1 (green) and PV (red) in the brain of 2-month-old male SARM1^f/f^ and SARM1^PV^-CKO mice. Cortex images were shown at a higher magnification. (F) Body weights of SARM1^f/f^ and SARM1^PV^-CKO mice at different developmental stages (n=6 animals per group). The red asterisk represents the specific band of SARM1. The density of the western blot bands was normalized to that of the β-actin protein. Data were presented as the mean ± SEM. (D) Quantitative data were analyzed using the Student’s t-test. (F) Quantitative data were analyzed using two-way ANOVA followed by Bonferroni’s post hoc tests, compared to the control group. *^**^p <* 0.01, *^***^p <* 0.001*.* Scale bars, 20 μm.


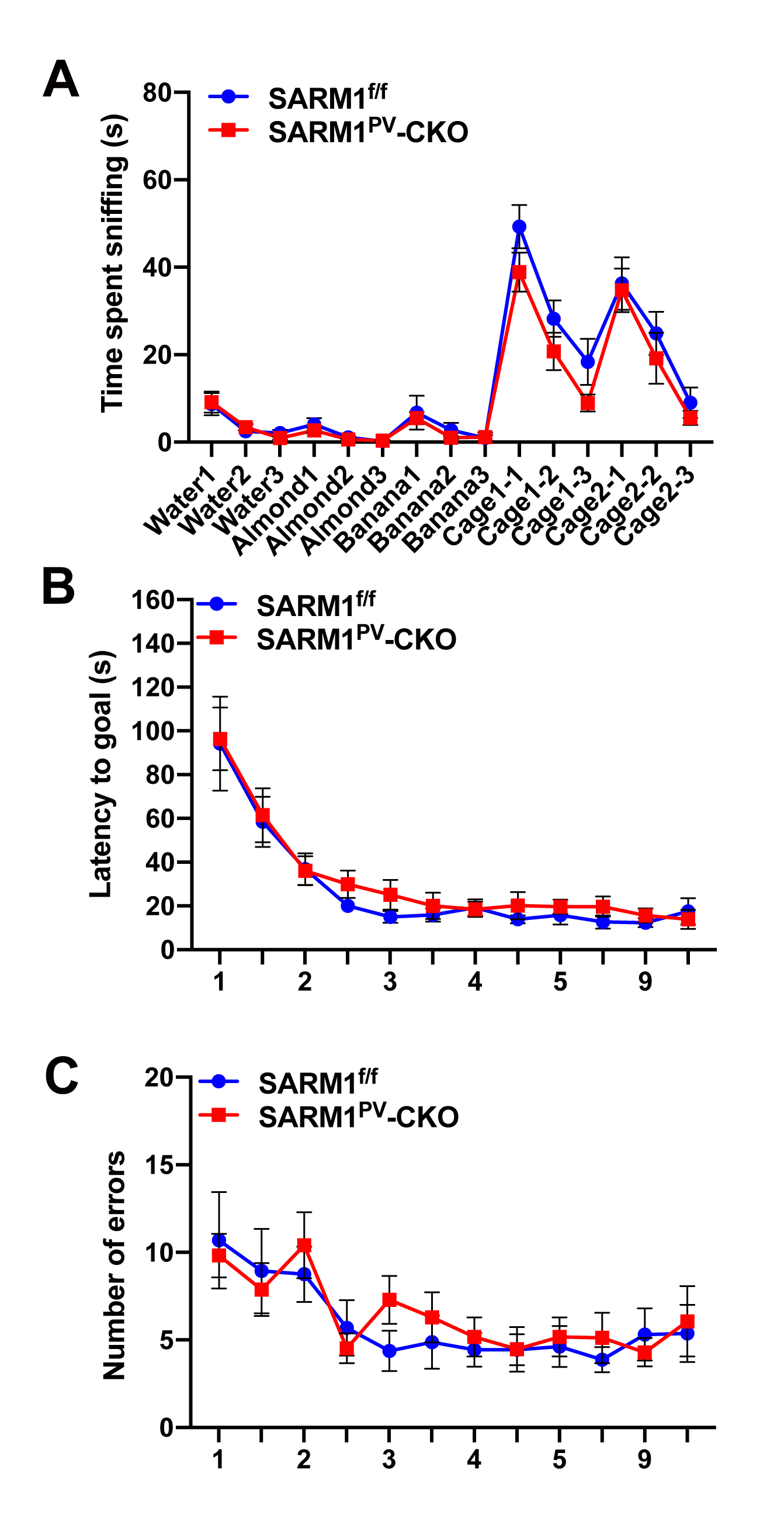


**Figure S2. SARM1^PV^-CKO male mice did not show cognitive dysfunction.** (A) Quantitative analysis of time spent sniffing in olfactory habituation/dishabituation test involving 2-month-old male SARM1^f/f^ (n=16 animals per group) or SARM1^PV^-CKO mice (n=17 animals per group). (B) Quantitative analysis of latency to goal in Barnes Maze test of 2-month-old male SARM1^f/f^ (n=16 animals per group) or SARM1^PV^-CKO mice (n=17 animals per group). (C) Quantitative analysis of number of errors in Barnes Maze test of 2-month-old male SARM1^f/f^ (n=16 animals per group) or SARM1^PV^-CKO mice (n=17 animals per group). Data were presented as the mean ± SEM. (A) Quantitative data were analyzed using the Student’s t-test. (B, C) Quantitative data were analyzed using two-way ANOVA followed by Bonferroni’s post-hov test, compared to the SARM1^f/f^ group.

**
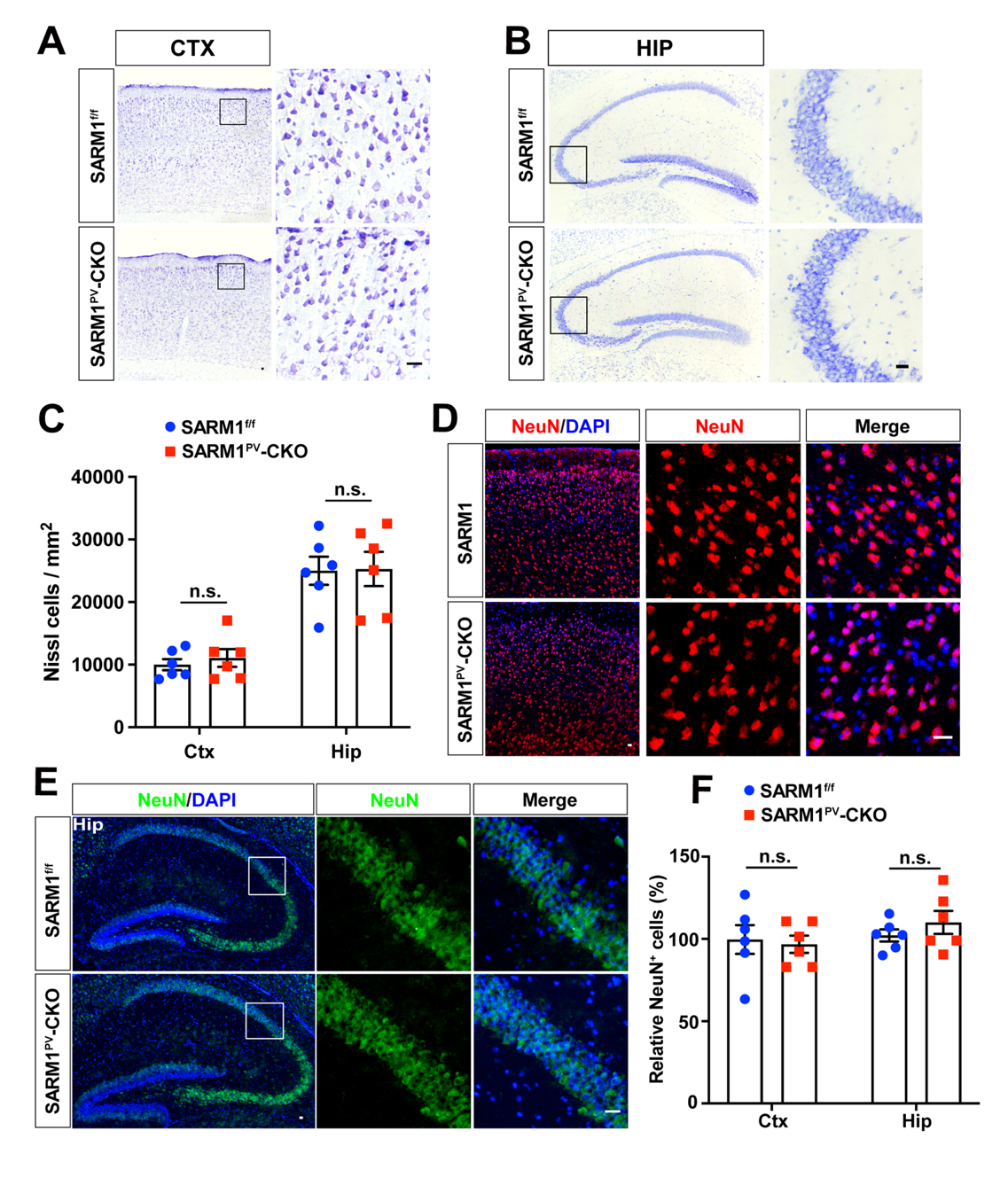
**

**Figure S3. Normal neuron development in cortex and hippocampus of SARM1^PV^-CKO male mice.** (A-B) Nissl staining images of the cortex (A) or hippocampus (B) obtained from 2-month-old male SARM1^f/f^ and SARM1^PV^-CKO mice. (C) Quantitative analysis of Nissl positive cells counts in different brain regions as shown in (A-B) (n=6 animals per group). (D-E) Immunostaining of NeuN in the cortex (D) or hippocampus (E) of 2-month-old male SARM1^f/f^ and SARM1^PV^-CKO mice. (F) Quantitative analysis of relative NeuN^+^ cells in different brain regions as shown in (D-E) (n=6 animals per group). Data were presented as the mean ± SEM. Quantitative data were analyzed using the Student’s t-test, compared to the control group. Scale bars, 20 μm.


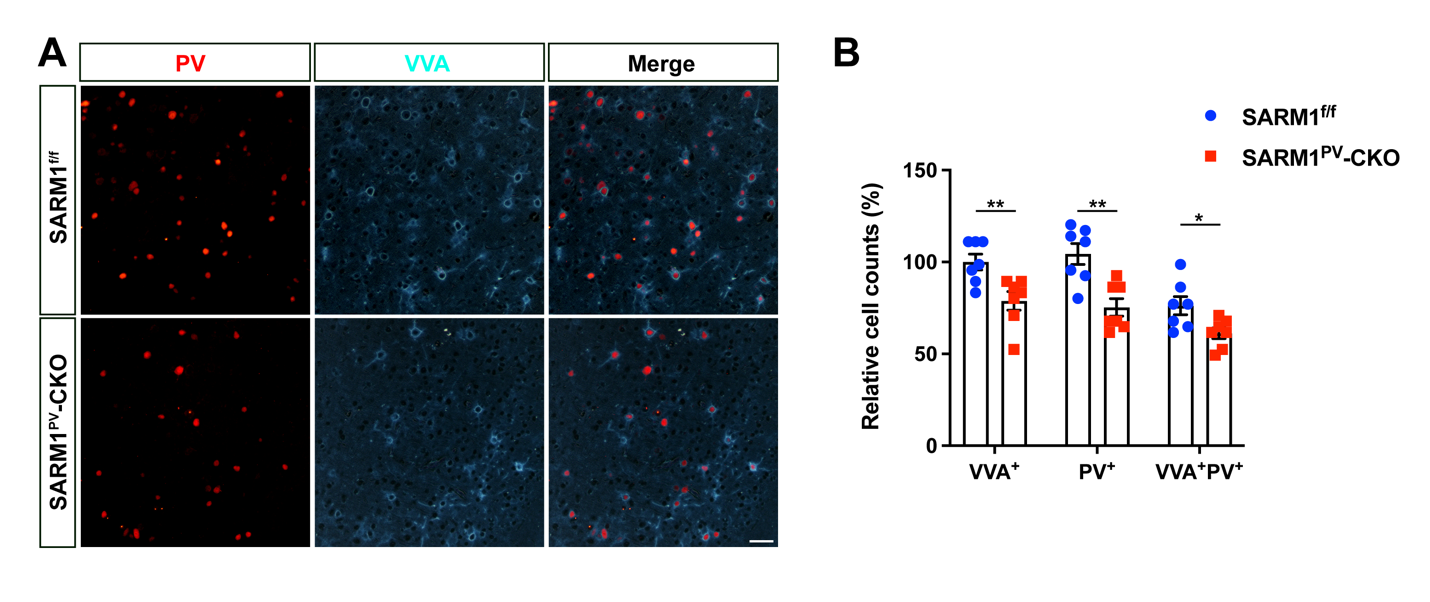


**Figure S4. Double-labelled PVIs were decreased in the cortex of SARM1^PV^-CKO mice.** (A) Immunostaining of PV (red) and VVA (DAB staining, pseudo-colored cyan) in mice cortex of SARM1^f/f^ and SARM1^PV^-CKO mice. (B) Quantitative analysis of the number of VVA^+^, PV^+^, or VVA^+^PV^+^ cells as shown in (A) (n=7 animals per group). Data were presented as the mean ± SEM. Quantitative data were analyzed using the Student’s t test, compared to the control group. *^*^p <* 0.05, *^**^p <* 0.01*.* Scale bars, 50 μm.

**
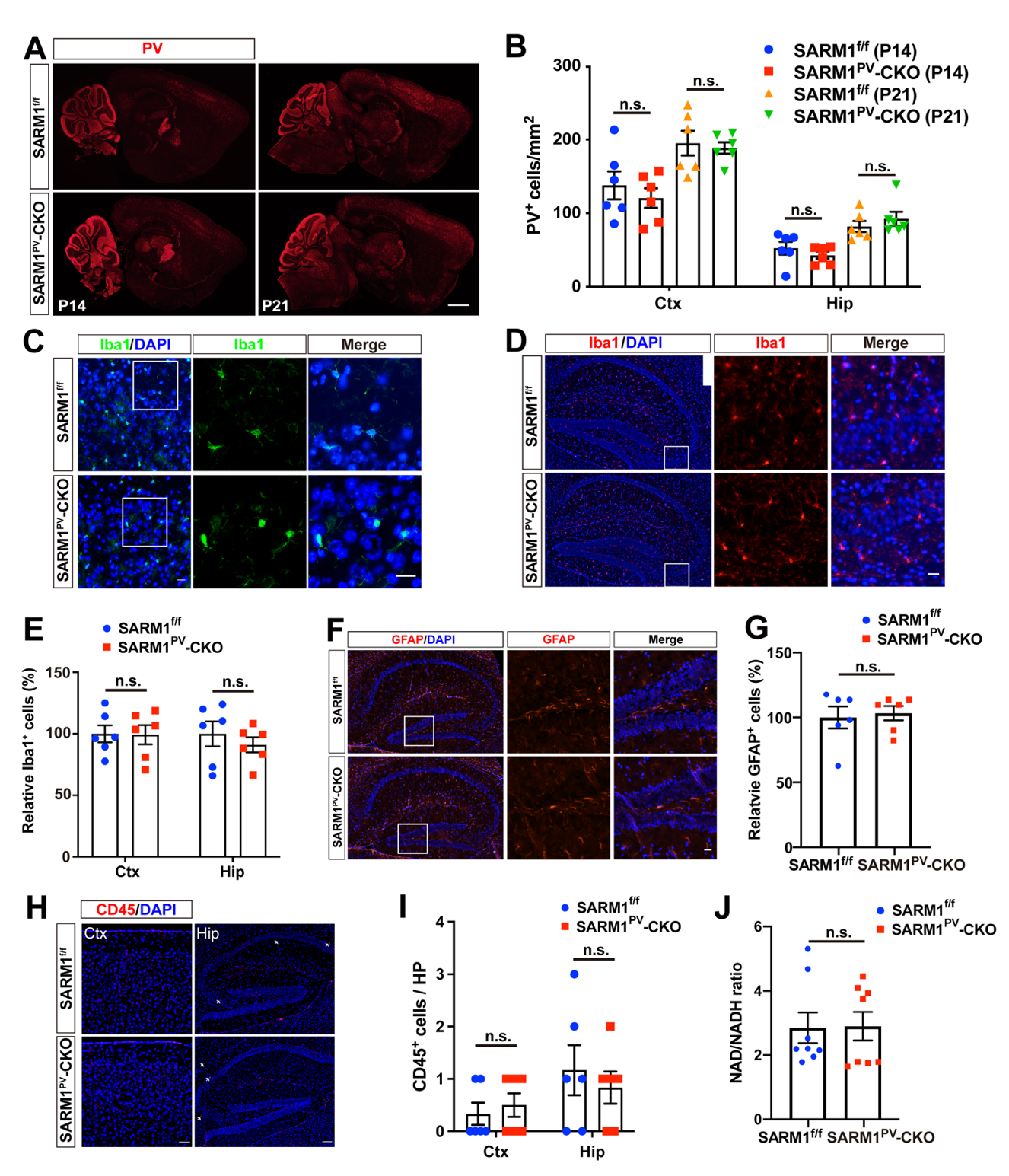
**

**Figure S5. SARM1 knockout in PVIs did not affect the development of PVIs and initiate inflammatory responses.** (A) Immunostaining of PV (red) in mice brain tissues of SARM1^f/f^ and SARM1^PV^-CKO mice at P14 and P21. (B) Quantitative analysis of the number of PV^+^ cells in the cortex and hippocampus per mm^2^ as shown in (A) (n=6 animals per group). (C-D) Immunostaining of Iba1 in the cortex (C) or hippocampus (D) of 2-month-old male SARM1^f/f^ and SARM1^PV^-CKO mice. Images of selected regions (white squares) were shown at a higher magnification. (E) Quantitative analysis of relative Iba1^+^ cells in different brain regions as shown in (C-D) (n=6 animals per group). (F) Immunostaining of GFAP (red) in the hippocampus of 2-month-old male SARM1^f/f^ and SARM1^PV^-CKO mice. Images of selected regions (white squares) were shown at a higher magnification. (G) Quantitative analysis of relative GFAP^+^ cells in brain regions as shown in (F) (n=6 animals per group). (H) Immunostaining of CD45 (red) in the cortex and hippocampus of 2-month-old male SARM1^f/f^ and SARM1^PV^-CKO mice. (I) Quantitative analysis of CD45^+^ cells in brain regions as shown in (H) (n=6 animals per group). (J) Quantitative analysis of NAD/NADH ratio in prefrontal cortex (n=8 animals per group). Quantitative data were analyzed using the Student’s t test, compared to the control group. (A) Scale bars, 1 mm. (C, D, F) Scale bars, 20 μm. (H) Scale bars, 50 μm.
