# Supplementary material for: SARM1 deletion in parvalbumin neurons is associated with autism-like behaviors in mice
Source: Cell Death Dis. 2022 Jul 22;13(7):638. doi: 10.1038/s41419-022-05083-2 (PMC9307765; doi:10.1038/s41419-022-05083-2)
Supplement: Supplementary file 3 — Original western blots [file 41419_2022_5083_MOESM3_ESM.docx]

**Original data of western blot**


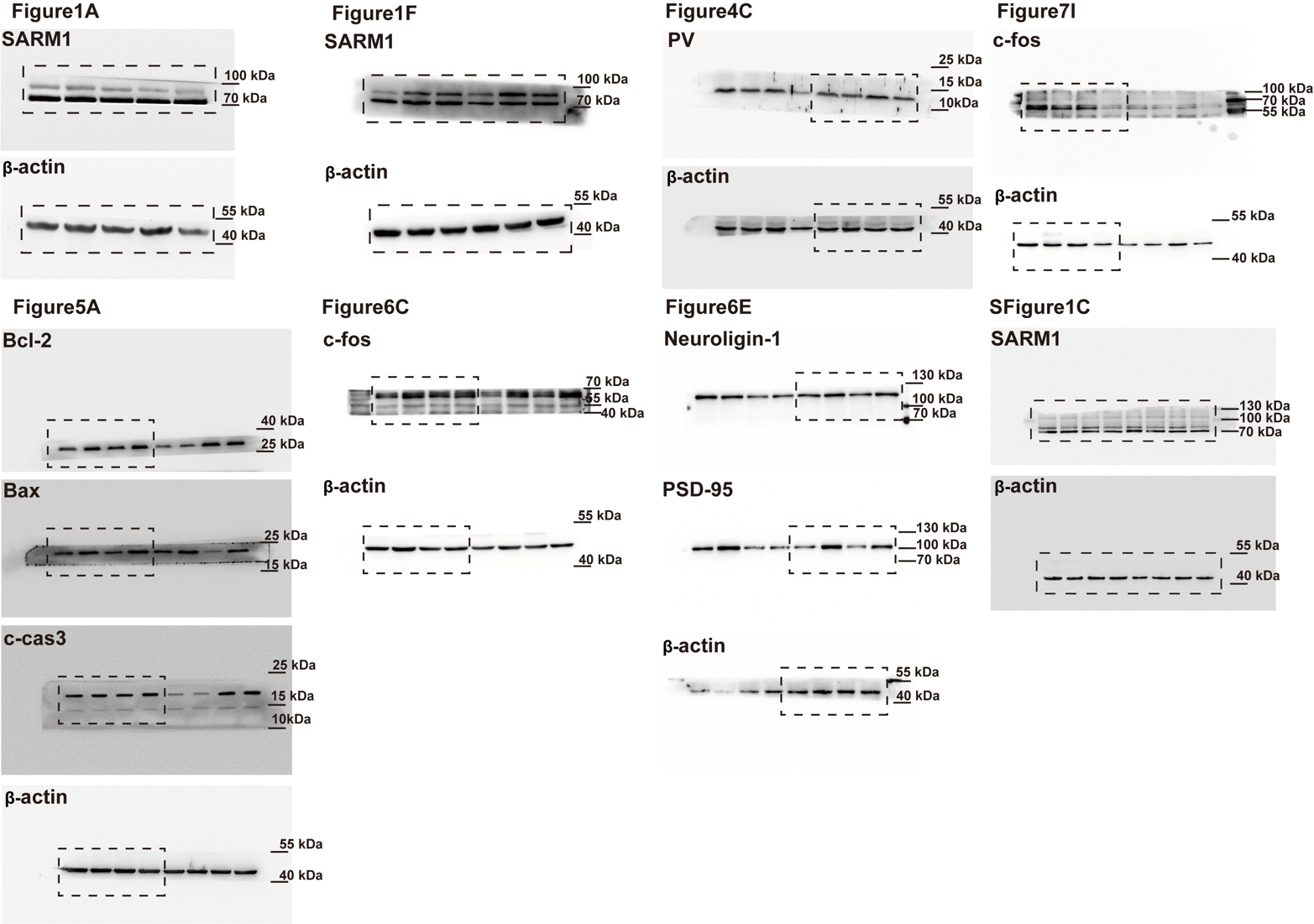


Protein was separated by SDS-PAGE (8%–12%) and transferred into nitrocellulose (G9944566, GE Amersham) or polyvinylidene fluoride membranes (ISEQ10100, Millipore). Membranes covered molecular weight of the target protein were indicated by the markers, and incubate with the corresponding primary antibody.
